# Supplementary material for: A motogenic GABAergic system of mononuclear phagocytes facilitates dissemination of coccidian parasites
Source: eLife. 2020 Nov 12;9:e60528. doi: 10.7554/eLife.60528 (PMC7685707; doi:10.7554/eLife.60528)
Supplement: Supplementary file 1. [file elife-60528-supp1.docx]

**Supplementary File 1** Primer pair sequences used in real-time quantitative PCR.

| **mouse mRNA target** | **Forward primer sequence** | **Reverse primer sequence** | **Amplicon** |
| --- | --- | --- | --- |
| Enzymes |  |  |  |
| GAD65 (*Gad2* ) | GCTGGAACCACCGTGTATGG | TCCACGTGCATCCAGATCTTAT | 86 |
| GAD67 (*Gad1* ) | GTGACCAGGGTGCCCGCTTC | TGCGCAGTTTGCTCCTCCCC | 100 |
| GABA-T (*Abat* ) | TGGCCTTCTTGTTGATTACC | TATATCTGGATCCAGGTATGAAGAG | 81 |
| GABA-A R subunits |  |  |  |
| ⍺1(*GabrA1* ) | AAAAGCGTGGTTCCAGAAAA | GCTGGTTGCTGTAGGAGCAT | 84 |
| ⍺2 (*GabrA2* ) | GCTACGCTTACACAACCTCAGA | GACTGGCCCAGCAAATCATACT | 115 |
| ⍺3 (*GabrA3* ) | GCCGTCTGTTATGCCTTTGTATTT | TTCTTCATCTCCAGGGCCTCT | 119 |
| ⍺4 (*GabrA4* ) | AGAACTCAAAGGACGAGAAATTGT | TTCACTTCTGTAACAGGACCCC | 118 |
| ⍺5 (*GabrA5* ) | GATTGTGTTCCCCATCTTGTTTGGC | TTACTTTGGAGAGGTGGCCCCTTTT | 100 |
| ⍺6 (*GabrA6* ) | TGGGAGCTATGCTTATCCT | TCTTCTGGGACTTCTACTGAG | 81 |
| β1 (*GabrB1* ) | GGTTTGTTGTGCACACAGCTCC | ATGCTGGCGACATCGATCCGC | 153 |
| β2 (*GabrB2* ) | GCTGGTGAGGAAATCTCGGTCCC | CATGCGCACGGCGTACCAAA | 70 |
| β3 (*GabrB3* ) | GAGCGTAAACGACCCCGGGAA | GGGACCCCCGAAGTCGGGTCT | 100 |
| γ1 (*GabrG1* ) | ATCCACTCTCATTCCCATGAACAGC | ACAGAAAAAGCTAGTACAGTCTTTGC | 100 |
| γ2 (*GabrG2* ) | ACTTCTGGTGACTATGTGGTGAT | GGCAGGAACAGCATCCTTATTG | 147 |
| γ3 (*GabrG3* ) | ATTACATCCAGATTCCACAAGATG | CAC AGG TGT CCT CAA ATT CCT | 149 |
| δ (*GabrD*) | GAATCCGTTCCAGACTCAAA | GCACTAGGCTCAACTTCAGG | 349 |
| 𝜀 (*GabrE*) | ACTGCGCCCTGGCATTGGAG | AGGCCCGAGGCTGTTGACAA | 70 |
| θ (*GabrQ* ) | GCTGGAGGTGGAGAGCTATGGCT | CCCCAGGTACGTGTACTGAGGGA | 115 |
| π (*GabrP* ) | CAGAGGACGTGCATCCAGGGGA | TCCGAACTGGGTCACCACCGAA | 139 |
| ⍴1 (*GabrR1* ) | CTGGAAATCGAAAGCTACGC | AGATGTGACGACGCAGAGTG | 205 |
| ⍴2 (*GabrR2* ) | CCAAGCCAAGCCATTTGTAT | GTCCCTCCAGTAATGCCTCA | 227 |
| ⍴3 (*GabrR3* ) | CAACTCAACAGGAGGGGAAA | TCCACATCAGTCTCGCTGTC | 101 |
| Transporters |  |  |  |
| GAT1 (*Slc6a1* ) | TGAACTCTTCATTGCTGCC | AAGACATAAATGCCACCCTG | 81 |
| GAT2/BGT1 (*Slc6a12* ) | CATGATGCCTTTGTCCCAG | TACAGACGAACTGGCTGTC | 83 |
| GAT3 (*Slc6a13* ) | ATCTTTGAAGGCATCGGCT | GAAGAGGTAGAAGAGGG | 96 |
| GAT4 (*Slc6a11* ) | Mm_Gabt4_1_SG QuantiTect Primer Assay | (QT00130116, Quiagen) | 78 |
| CCCs |  |  |  |
| NKCC1 (*Slc12a2* ) | GATGCTGTGGTCGCATACACT | CAGCGGACTAATACACCCTTG | 77 |
| NKCC2 (*Slc12a1* ) | CTGGCTAAGAATGTGACTGT | TCTGCTTGCTCATCTCCAG | 83 |
| KCC1 (*Slc12a4* ) | TCTACCTGGGGACGACATTTG | CCGATGGGTAAAAGATGGCAG | 102 |
| KCC2 (*Slc12a5* ) | TCAGTCACAGGGATCATGG | GGATAGTTCCAGTAGGGATAGAC | 82 |
| KCC3 (*Slc12a6* ) | CTGCCATCTTTCGGAGTGACG | AGAAGGCTGTACCATAGACGC | 81 |
| KCC4 (*Slc12a7* ) | ATGCCCACGAACTTTACGGTG | GGGAGGTTTGATCCACGCT | 115 |
| VDCCs |  |  |  |
| CaV 1.1 (*Cacna1S* ) | GGTAGCATGTAAGAGGCTG | GCAAATAGTGTGGCATTAAAGG | 81 |
| CaV 1.2 (*Cacna1C*) | CGTTCTCATCCTGCTCAACA | TATGCTCCCAATGACGATGA | 208 |
| CaV 1.3 (*Cacna1D*) | TGCACAGATGAAGCCAAAAG | GACCAACGTTCTCACCGTTT | 229 |
| CaV 1.4 (*Cacna1F* ) | TCCATCATGAAGGCGCTTGT | CGAGTCCGATGATGGCGTAA | 85 |

| CaV 2.1 (*Cacna1A* ) | AATTCCAAATCACGGAGCAC | CATCAGAAACGAGCACAGGA | 218 |
| --- | --- | --- | --- |
| CaV 2.2 (*Cacna1B* ) | GCAACACATGGAACTGGTTG | GCATTCTTGTCCTCCTCTGC | 238 |
| CaV 2.3 (*Cacna1E* ) | TGAAGGCTGTGTTTGACTGC | ATTCATGACGCTTCCATTCC | 234 |
| CaV 3.1 (*Cacna1G* ) | TGTGGAAATGGTGGTGAAGA | ACTGCGGAGAAGCTGACATT | 150 |
| CaV 3.2 (*Cacna1H*) | TGGGAACGTGCTTCTTCTCT | GGGGATGTGTGAGCATTTCT | 229 |
| Reference genes TATA-binding protein |  |  |  |
| (*TBP* ) | GGGGAGCTGTGATGTGAAGT | CCAGGAAATAATTCTGGCTCA | 93 |
| Importin 8 (*IPO8* ) | CTATGCTCTCGTTCAGTATGC | GTCCGAAAGATCTCCATCCA | 81 |
| Beta-Actin (*Actb* ) | CACTGTCGAGTCGCGTCC | TCATCCATGGCGAACTGGTG | 89 |
| Glyceraldehyde-3- |  |  |  |
| phosphate |  |  |  |
| dehydrogenase |  |  |  |
| (*GAPDH*) | TGACCTCAACTACATGGTCTACA | CTTCCCATTCTCGGCCTTG | 85 |
|  |  |  |  |
| **human mRNA target** | **Forward primer sequence** | **Reverse primer sequence** | **Amplicon** |
| Enzymes |  |  |  |
| GAD65 (*Gad2* ) | CTACGCGTTTCTCCATGCAA | GCCAAAGTGGGCCTTTCTC | 63 |
| GAD67 (*Gad1* ) | GCGGACCCCAATACCACTAAC | CACAAGGCGACTCTTCTCTTC | 144 |
| GABA-T (*Abat* ) | TGAAATACCCTCTGGAAGAG | CAATCAGATCCTCCACCTC | 80 |
| GABA-A R subunits |  |  |  |
| ⍺1(*GabrA1* ) | GTCACCAGTTTCGGACCCG | AACCGGAGGACTGTCATAGGT | 119 |
| ⍺2 (*GabrA2* ) | GTTCAAGCTGAATGCCCAAT | ACCTAGAGCCATCAGGAGCA | 160 |
| ⍺3 (*GabrA3* ) | CAACTTGTTTCAGTTCATTCATCCTT | CTTGTTTGTGTGATTATCATCTTCTTAGG | 102 |
| ⍺4 (*GabrA4* ) | TTGGGGGTCCTGTTACAGAAG | TCTGCCTGAAGAACACATCCA | 105 |
| ⍺5 (*GabrA5* ) | TTGGATGGCTACGACAACAGA | GTCCTCACCTGAGTGATGCG | 62 |
| ⍺6 (*GabrA6* ) | ACCCACAGTGACAATATCAAAAGC | GGAGTCAGGATGCAAAACAATCT | 67 |
| β1 (*GabrB1* ) | TGCATGTATGATGGATCTTCG | GTGGTATAGCCATAACTTTCGA | 80 |
| β2 (*GabrB2* ) | GCAGAGTGTCAATGACCCTAGT | TGGCAATGTCAATGTTCATCCC | 137 |
| β3 (*GabrB3* ) | CAAGCTGTTGAAAGGCTACGA | ACTTCGGAAACCATGTCGATG | 108 |
| γ1 (*GabrG1* ) | CCTTTTCTTCTGCGGAGTCAA | CATCTGCCTTATCAACACAGTTTCC | 91 |
| γ2 (*GabrG2* ) | CACAGAAAATGACGGTGTGG | TCACCCTCAGGAACTTTTGG | 136 |
| γ3 (*GabrG3* ) | AACCAACCACCACGAAGAAGA | CCTCATGTCCAGGAGGGAAT | 113 |
| δ (*GabrD*) | CTTTGCTCATTTCAACGCC | TTCCTCACGTCCATCTCTG | 86 |
| 𝜀 (*GabrE*) | ACAGGAGTGAGCAACAAAACTG | TGAAAGGCAACATAGCCAAA | 107 |
| θ (*GabrQ* ) | CCAGGGTGACAATTGGCTTAA | CCCGCAGATGTGAGTCGAT | 63 |
| π (*GabrP* ) | CAATTTTGGTGGAGAACCCG | GCTGTCGGAGGTATATGGTG | 110 |
| ⍴1 (*GabrR1* ) | GAAAGGCAGCCCAATTCTG | TCTATCCTCAGAAGCTGTTCTG | 80 |
| ⍴2 (*GabrR2* ) | TACAGCATGAGGATTACGGT | CAAAGAACAGGTCTGGGAG | 81 |
| ⍴3 (*GabrR3* ) | TGATGCTTTCATGGGTTTCA | CGCTCACAGCAGTGATGATT | 111 |
| Transporters |  |  |  |
| GAT1 (*Slc6a1* ) | TTTCTGTGCCTGTTACCCA | ATGTCTCGGAATTTGGACG | 126 |
| GAT2 (*Slc6a13* ) | CTTCACTAAGGTGGGATGGA | TTCCATGACTGGATACACTGG | 81 |
| GAT3 (*Slc6a11* ) | TATTTGAAGGCATTGGCTATGC | GTAGTGAAGCAGTTGCTCAG | 108 |
| BGT1 (*Slc6a12* ) | ACGACCTGCAACAACTTTTGG | GCTCCTGAGTGGTTCAGAAAGTC | 62 |

| CCCs |  |  |  |
| --- | --- | --- | --- |
| NKCC1 (*Slc12a2* ) | TGGGTCAAGCTGGAATAGGTC | ACCAAATTCTGGCCCTAGACTT | 161 |
| NKCC2 (*Slc12a1* ) | TCAGGAGATTTGGAGGATCCC | ACCCCTAAGTAGGCAACAGTG | 86 |
| KCC1 (*Slc12a4* ) | CCTCCCGTGTTTCCGGTATG | CAGGAGTCGGTCGTAAGGTTG | 155 |
| KCC2 (*Slc12a5* ) | GGAAGGAAATGAGACGGTGA | TCCCACTCCTCTCCACAATC | 200 |
| KCC3 (*Slc12a6* ) | GGATGTCATCGAGGACCTGAG | TCGAGCTTTCTTATGTCCGTC | 82 |
| KCC4 (*Slc12a7* ) | ATCTACTTCCCTTCCGTGACC | TCTGTGCATCCTTGAGGTCC | 70 |
| VDCCs |  |  |  |
| CaV 1.1 (*Cacna1S* ) | TGTCATCCTCAGTGAGATCG | TCATCTGGGTCAACGTTCC | 90 |
| CaV 1.2 (*Cacna1C*) | TTCCAACCTGGAACGAGTGG | AGGCATTGGGGTGAAAGAGG | 101 |
| CaV 1.3 (*Cacna1D*) | GGTGATCCCCTTCCCCATTC | ATAGTTTGCCTCGTTCGCGT | 162 |
| CaV 1.4 (*Cacna1F* ) | CCTGGGATCCGACATGGAAG | ACTCAGTCTGGTTCAGCGTG | 89 |
| CaV 2.1 (*Cacna1A* ) | TTCAACATCGTCTTCACCTC | GCGGAAATAATTCAGAATCCC | 81 |
| CaV 2.2 (*Cacna1B* ) | GGAATGTCTTTGACTTTGTCAC | TGAAATTGTTCGTTTCCGC | 81 |
| CaV 2.3 (*Cacna1E* ) | GATGGGACTCCTTCGGCAAA | CCCGTCAGGATCTGGAACAC | 71 |
| CaV 3.1 (*Cacna1G* ) | CCACGTGGTCCTTGTCATCA | GGGTCAGGAAGATGCGTTCA | 98 |
| CaV 3.2 (*Cacna1H*) | CATCTTCCAGGTGATCACG | ATGAAGTTGTAGAATGAGTGGG | 81 |
| CaV 3.3 (*Cacna1I* ) | GTGGAGATCATGTACTACGTG | GGAGCCCACTATGATAAGC | 81 |
| Reference genes TATA-binding protein (*TBP* ) | GAGCTGTGATGTGAAGTTTCC | TCTGGGTTTGATCATTCTGTAG | 117 |
| Importin 8 (*IPO8* ) | GCAAAGGAAGGGGAATTGAT | CGAAGCTCACTAGTTTTGACCC | 91 |
|  |  |  |  |
| ***T. gondii* target** | **Forward primer sequence** | **Reverse primer sequence** | **Amplicon** |
| *B1* | GCATTGCCCGTCCAAACT | AGACTGTACGGAATGGAGACGAA | 61 |
| *AMA1* | TGGAGAGAACCCAGATGCGTTCCT | CAGTGTAGTCGAGGCAACGGCC | 101 |

Primer pair sequences are designed using GETprime [1] (http://bbcftools.epfl.ch/getprime) or NCBI primer blast database (https://www.ncbi.nlm.nih.gov/tools/primer-blast/).

1. David FP, Rougemont J, Deplancke B. GETPrime 2.0: gene- and transcript-specific qPCR primers for 13 species including polymorphisms. Nucleic acids research. 2017;45(D1):D56-D60. Epub 2017/01/06. doi: 10.1093/nar/gkw913. PubMed PMID: 28053161; PubMed Central PMCID: PMCPMC5210624.
