# Supplementary material for: A motogenic GABAergic system of mononuclear phagocytes facilitates dissemination of coccidian parasites
Source: eLife. 2020 Nov 12;9:e60528. doi: 10.7554/eLife.60528 (PMC7685707; doi:10.7554/eLife.60528)
Supplement: Supplementary file 2. [file elife-60528-supp2.docx]

**Supplementary File 2** GABA-A R subunits and VDCCs transcribed by phagocytes.

| **Cell type** | **Challenge** | **α1** | **α2** | **α3** | **α4** | **α5** | **α6** | **β1** | **β2** | **β3** | **γ1** | **γ2** | **γ3** | **δ** | **ε** | **θ** | **π** | **ρ1** | **ρ2** | **ρ3** |
| --- | --- | --- | --- | --- | --- | --- | --- | --- | --- | --- | --- | --- | --- | --- | --- | --- | --- | --- | --- | --- |
| mBMDCs | Unchallenged |  |  | X | X | X |  |  | X | X | X | X |  | X |  |  |  | X | X |  |
|  | *T. gondii*-infected |  |  | X | X | X |  |  | X | X | X | X |  | X |  |  |  | X | X |  |
| hMoDCs | Unchallenged |  |  | 1/3* | X |  |  | 1/3 |  | 1/3 |  |  |  |  |  |  |  | X | X | X |
|  | *T. gondii*-infected |  |  | 1/3 | X |  | X | 2/3 | X | 2/3 |  |  |  |  |  | X |  | 1/3 | X | X |
| hMDCs | Unchallenged |  |  |  |  |  | 2/7 | 1/7 | X |  |  |  |  |  |  | X |  | X | X |  |
|  | *T. gondii*-infected |  |  |  |  |  | 3/7 | X | X |  |  |  |  |  |  | X |  | X | X |  |
| hMonocytes | Unchallenged | 1/3 |  |  | X |  | X |  |  |  | X |  |  |  |  |  |  |  | X |  |
|  | *T. gondii*-infected | 2/3 |  |  | X |  | X |  | 2/3 |  | X |  |  |  |  |  |  |  | X |  |

X – all samples expressed the particular mRNA

* – number of donors expressing the particular mRNA related to total number of donors tested

| **Cell type** | **Challenge** | **Ca_V_1.1** | **Ca_V_1.2** | **Ca_V_1.3** | **Ca_V_1.4** | **Ca_V_2.1** | **Ca_V_2.2** | **Ca_V_2.3** | **Ca_V_3.1** | **Ca_V_3.2** | **Ca_V_3.3** |
| --- | --- | --- | --- | --- | --- | --- | --- | --- | --- | --- | --- |
| mBMDCs^a^ | Unchallenged | X | X | X | X | X | X | X | X | X | Not tested |
|  | *T. gondii*-infected | X | X | X | X | X | X | X | X | X | Not tested |
| hMoDCs | Unchallenged |  | X | X | X | X | X | X | X | 2/3* | X |
|  | *T. gondii*-infected |  | X | X | X | X | X | X | X | 1/3 | X |
| hMDCs | Unchallenged |  |  |  | X |  |  | 2/7 | X |  |  |
|  | *T. gondii*-infected |  |  |  | X |  |  | 4/7 | X |  |  |
| hMonocytes | Unchallenged |  |  | X | X |  |  |  |  |  |  |
|  | *T. gondii*-infected |  |  | X | X |  |  |  |  |  |  |

a – Kanatani et al., 2017

X – all samples expressed the particular mRNA

* – number of donors expressing the particular mRNA related to total number of donors tested
