# Supplementary material for: A motogenic GABAergic system of mononuclear phagocytes facilitates dissemination of coccidian parasites
Source: eLife. 2020 Nov 12;9:e60528. doi: 10.7554/eLife.60528 (PMC7685707; doi:10.7554/eLife.60528)
Supplement: Supplementary file 3. [file elife-60528-supp3.docx]

**Supplementary File 3** Sh-RNA construct sequences.

| **Target** | **Sequence (5' to 3') RestrictionSite-SENSE (Target sequence)-LOOP-ANTISENSE-PolyT-C or G** | **Cloning Vector** | **Source** |
| --- | --- | --- | --- |
| Luciferase (non-existent in mouse and human) | TG-TTCTCCGAACGTGTCACGT-TTCAAGAGA-ACGTGACACGTTCGGAGAA-CTTTTTTC | pLL3.7 | Varas-Godoy lab |
| mouse *Gabra3*_1 (GABA-A R subunit *a*3) | TG-TGGGTACCACCTATCCTATCAAT-TTCAAGAGA-ATTGATAGGATAGGTGGTACCCA-CTTTTTTC | pLL3.7 | Varas-Godoy lab |
| mouse *Gabra3*_2 (GABA-A R subunit *a*3) | CCGG-GGGAATCAAGACGACAAGAAC-CTCGAG-GTTCTTGTCGTCTTGATTCCC-TTTTTG | pLKO.1 | Sigma-Aldrich |
| mouse *Gabrb3* (GABA-A R subunit *b*3) | CCGG-CCCTGATCTAACCGATGTGAA-CTCGAG-TTCACATCGGTTAGATCAGGG-TTTTTG | pLKO.1 | Varas-Godoy lab |
| mouse *Gabrr1* (GABA-A R subunit *r*1) | CCGG-GACGCTATATTTGAGGCACTA-CTCGAG-TAGTGCCTCAAATATAGCGTC-TTTTTG | pLKO.1 | Sigma-Aldrich |
| mouse *Slc12a2* (NKCC1) | CCGG-GCCGAGAGTAAAGGAGTTGTA-CTCGAG-TACAACTCCTTTACTCTCGGC-TTTTTG | pLKO.1 | Sigma-Aldrich |
| human *SLC12A2* (NKCC1) | TG-GCCAAATATCAGCGATGGCTT-TTCAAGAGA-AAGCCATCGCTGATATTTGGC-CTTTTTTC | pLL3.7 | Genscript |
| human *GAD1* (GAD67) | TG-GCTCTCCACTGGATTGGATAT-TTCAAGAGA-ATATCCAATCCAGTGGAGAGC-CTTTTTTC | pLL3.7 | Genscript |
| human *GABRA4* (GABA-A R subunit *a*4) | TG-CGGGAGTTATGCCTATCCAAA-TTCAAGAGA-TTTGGATAGGCATAACTCCCG-CTTTTTTC | pLL3.7 | Genscript |
| human *GABRR2* (GABA-A R subunit *r*2) | TG-GTCTTCTTTGTTCACTCCAAA-TTCAAGAGA-TTTGGAGTGAACAAAGAAGAC-CTTTTTTC | pLL3.7 | Genscript |
| human *CACNA1D* (CaV1.3) | TG-CCAAGCAAACTGTCCTGTCTT-TTCAAGAGA-AAGACAGGACAGTTTGCTTGG-CTTTTTTC | pLL3.7 | Genscript |
